# Iron Metabolism in Aging and Age-Related Diseases

**DOI:** 10.3390/ijms23073612

**Published:** 2022-03-25

**Authors:** Yao Tian, Yuanliangzi Tian, Zhixiao Yuan, Yutian Zeng, Shuai Wang, Xiaolan Fan, Deying Yang, Mingyao Yang

**Affiliations:** 1 Institute of Animal Genetics and Breeding, Sichuan Agricultural University, Chengdu 611130, China; tianyao0210@163.com (Y.T.); 2575026t@student.gla.ac.uk (Y.T.); yzx15699336321@163.com (Z.Y.); alisaz1996@hotmail.com (Y.Z.); wangshuai36@outlook.com (S.W.); xiaolanfan@sicau.edu.cn (X.F.); dnaydy@126.com (D.Y.); 2 Farm Animal Genetic Resources Exploration and Innovation Key Laboratory of Sichuan Province, Sichuan Agricultural University, Chengdu 611130, China

**Keywords:** iron metabolism, aging, mitochondria, neurodegenerative diseases, iron chelator

## Abstract

Iron is a trace metal element necessary to maintain life and is also involved in a variety of biological processes. Aging refers to the natural life process in which the physiological functions of the various systems, organs, and tissues decline, affected by genetic and environmental factors. Therefore, it is imperative to investigate the relationship between iron metabolism and aging-related diseases, including neurodegenerative diseases. During aging, the accumulation of nonheme iron destroys the stability of the intracellular environment. The destruction of iron homeostasis can induce cell damage by producing hydroxyl free radicals, leading to mitochondrial dysfunction, brain aging, and even organismal aging. In this review, we have briefly summarized the role of the metabolic process of iron in the body, then discussed recent developments of iron metabolism in aging and age-related neurodegenerative diseases, and finally, explored some iron chelators as treatment strategies for those disorders. Understanding the roles of iron metabolism in aging and neurodegenerative diseases will fill the knowledge gap in the field. This review could provide new insights into the research on iron metabolism and age-related neurodegenerative diseases.

## 1. Introduction

Iron is the most abundant transition metal in humans [1], which is distributed in almost all tissues and organs, such as the liver, spleen, kidney, heart, skeletal muscle, and brain. Iron exists in many forms: one is functional iron existing in hemoglobin, myoglobin, enzymes, and cofactors, and another is reserve iron existing in ferritin and hemosiderin [2]. Iron is the auxiliary group of many key enzymes and participates in a series of important physiological processes such as oxygen transport, DNA synthesis and repair, and mitochondrial function maintenance [3]. Iron deficiency and iron overload will cause different degrees of harm to the organism. For example, low concentrations of iron result in consequent anemia [4]. By contrast, high concentrations of labile iron are highly toxic to cells increasing the risk of cancer, diabetes, neurodegenerative diseases, and cardiovascular diseases [5]. Importantly, iron overload can also cause a new type of iron-dependent cell death—Ferroptosis [6]. Thus, iron can act as a double-edged sword, which necessitates accurate regulation of its cellular levels and exquisite equilibrium between iron absorption, circulation, storage, and regulation.

Aging is characterized by a progressive loss of physiological integrity, leading to impaired function and increased vulnerability to death [7]. Currently, there are many coexisting theories about the hallmarks of aging, such as the telomere degradation theory, mitochondrial function damage theory, and oxidative damage theory [7]. Among them, the oxidative damage theory holds that reactive oxygen species (ROS) damage biological macromolecules such as lipids, proteins, and DNA, which leads to aging [8]. Mitochondria are the main source of ROS in cells. After reaching mitochondria, iron is mainly used to synthesize heme and iron–sulfur clusters [9]. Excessive iron has strong catalytic potential, which can increase ROS production and cause oxidative damage to cells, eventually leading to cellular senescence. A large number of studies have shown that the loss of mitochondrial iron homeostasis can result in mitochondrial function decline, and then lead to aging [10,11]. Thus, it can be seen that aging has a profound impact on iron homeostasis and it is necessary to elaborate on the complex interactions between iron homeostasis and aging.

Neurodegenerative diseases are caused by the loss of neurons and will worsen over time. As it stands now, iron is indeed a key factor in neurodegenerative diseases [12]. For instance, the imbalance of iron homeostasis in the brain may be at the core of many age-related neurodegenerative diseases such as Alzheimer’s disease and Parkinson’s disease [13,14]. Some less common neurodegenerative diseases, mainly including a series of neurodegeneration with brain iron accumulation (NBIA), have been widely studied and reported [15,16]. Following that, more and more iron chelators are being used to study the treatment of neurodegenerative diseases. This is just another imperative way to explore the potential therapeutic approaches for neurodegenerative diseases through the regulation of iron metabolism.

In this paper, we first describe the metabolic process of iron in the body. Secondly, we review the research advances between iron metabolism and aging and neurodegenerative diseases. Finally, we present some iron chelators as treatment strategies for the cure of age-related neurodegenerative diseases. It is hoped that this review can provide a deep understanding of the role of iron metabolism in aging and age-related neurodegenerative diseases.

## 2. Iron Metabolism

**Figure 1:**
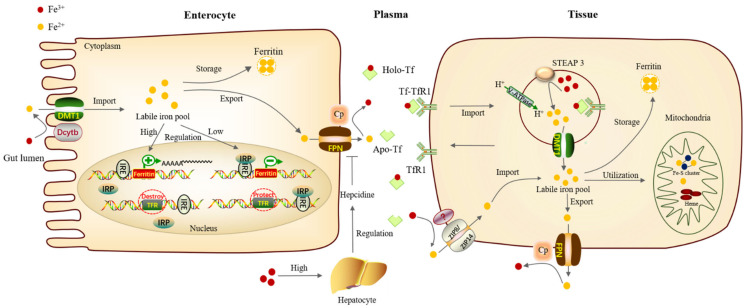
Iron metabolism process in mammals. The absorption, circulation, storage, and regulation of iron collaborate closely together to maintain the iron homeostasis of organisms. Firstly, Fe^3+^ in gut lumen is reduced into Fe^2+^ by Dcytb positioned on the parietal membrane of intestinal epithelial cells, then transported into the iron pool in the cytoplasm through DMT1. A part of iron forms ferritin for storage, and excess iron is transported out of the cells through FPN of the basal lateral membrane, then it is oxidized into Fe^3+^ by Cp. Fe^3+^ can combine with transferrin in plasma and transport to other tissues requiring iron through blood circulation. In tissues, after combining with transferrin receptor on the surface of the tissue needing iron, protein complex Tf-TfR1 enters cells through endocytosis. The acidification of early endosomes will change the conformation of Tf-TfR1 and promote the release of Fe^3+^. This acidification is mainly supported by the V-ATPase on the endosome through proton exchange. Fe^3+^ is then reduced to Fe^2+^ by STEAP3 and then enters cytoplasm through DMT1 for utilization. One part of the iron can be stored as ferritin, while other part of iron can enter the mitochondria for the synthesis of iron–sulfur clusters and heme, and the rest of iron is transported out of the cells through FPN. In iron deficiency, the IRPs bind to the IREs, protecting the TfR mRNA from nuclease digestion and preventing the synthesis of ferritin. When iron is abundant, the modified IRP no longer binds to the IREs, making TfR mRNA to be destroyed and allowing the expression of ferritin. Dcytb, Duodenal cytochrome b; DMT1, Divalent metal-ion transporter 1; FPN, Ferroportin; Cp, Ceruloplasmin; V-ATPase, vacuolar H^+^-ATPase; STEAP, Six-transmembrane epithelial antigen of prostate 3.

Maintaining safe iron levels is critical for sustaining iron homeostasis. However, under aerobic conditions, iron also has potential toxicity [17]. Controlling the absorption, circulation, storage, and regulation of iron at both the cellular and systemic levels limits the toxic effects of excessive or abnormal distribution of iron. Specific iron metabolism processes are governed by complex pathways (Figure 1).

In mammals, most of the iron ingested from food exists in the form of Fe^3+^ which is then reduced to Fe^2+^ by Duodenal cytochrome b (Dcytb) on the apical surface of intestinal epithelial cells. Fe^2+^ can be directly exported into the plasma through the Divalent metal-ion transporter 1 (DMT1) [18]. Besides, ZIP8/14 can also mediate iron absorption by acting as a cellular iron importer on the cell surface of a particular tissue [19,20]. Iron imported into the cell forms the cytoplasmic labile iron pool (LIP), which acts as an intermediate between imported, stored, and utilized iron [21]. The excess Fe^2+^ needs to be oxidized to Fe^3+^ by Hephaestin (Heph) and Ceruloplasmin (Cp), then be exported through the iron export protein, Ferroportin (FPN) [18].

Iron entering the circulatory system can combine with Transferrin (Tf) in plasma, then be transported to various tissues and organs hungry for iron in mammals. Transferrin carrying iron combines with transferrin receptor 1 (TfR1) on the cell membrane and is then internalized into the cell through endocytosis [22]. The acidification of early endosomes will change the conformation of the protein complex Tf-TfR1 and promote the release of Fe^3+^. This acidification is mainly supported by the vacuolar H^+^-ATPase (V-ATPase) on the endosome through proton exchange [23]. The six-transmembrane epithelial antigen of the prostate 3 (STEAP3) will reduce ferric Fe^3+^ to ferrous Fe^2+^, and then Fe^2+^ is released from the endosomes into the cytoplasm via DMT1 for utilization [24].

Some of the iron can be stored in the form of ferritin, while others can enter the mitochondria for the synthesis of iron–sulfur clusters and heme, and the excess iron is transported out of the cells through FPN [18]. In mammals, ferritin mainly exists in the cytoplasm, which is a hollow complex formed by light-chain homo (LCH) and heavy-chain homo (HCH). It can chelate about 4500 iron ions and is an important iron storage protein and detoxification protein [25]. Ferritin plays an important physiological role in maintaining iron homeostasis. For example, in *Drosophila*, reducing the levels of the ferritin heavy chain in the larval wing discs leads to drastic growth defects [26]. Moreover, in human metastatic melanoma cells, high ferritin expression can enhance cell growth and improve resistance to oxidative stress by interfering with their cellular antioxidant system [27].

In humans, efficient orchestration of iron absorption is critical for maintaining iron homeostasis. Hepcidin secreted by liver parenchymal cells is a key protein and the most important negative regulatory hormone for iron metabolism [28]. An increase in plasma and tissue iron levels stimulates hepcidin production in the liver and the levels of FPN protein on intestinal epithelial cells decrease, which results in inhibiting the absorption of iron in the intestine and a decrease in serum iron concentration [29]. On the other hand, cellular iron homeostasis is also controlled by influencing mRNA translation. Changes in iron status lead to compensating changes in the iron regulatory protein (IRP)/iron regulatory element (IRE) system of iron homeostasis. When iron concentrations are depleted, the IRPs bind to the IREs in the 3′- and 5′- untranslated regions (UTR), protecting the TfR mRNA from nuclease digestion and preventing the synthesis of ferritin in ferritin mRNA. Conversely, when there is surplus iron, the modified IRP no longer binds to the IREs, allowing TfR mRNA to be destroyed and allowing the expression of ferritin [30].

Regulation of iron homeostasis is an intricate process involving multiple modulators at the molecular, cellular, and systemic levels. Cross-talks between different levels are key to sensing iron levels, adjusting absorption, and recycling accordingly, which together maintain the iron homeostasis of the organism.

## 3. Iron Metabolism and Aging

Aging refers to the natural life process in which the physiological functions of various systems, organs, and tissues decrease due to the aging process, which is influenced by genetic factors and environmental factors [7]. In recent years, people have paid more attention to the role of iron ions in aging. Iron is a transition metal with two main biological oxidation states, and it is also a key intermediate for producing active oxygen [31]. Excessive accumulation of iron ions in aging cells produces excessive reactive oxygen species, which can lead to DNA damage and inhibit its repair function, subsequently accelerating the aging process, which is defined as Ferrosenescence [32].

### 3.1. Iron and the Production of ROS

**Figure 2:**
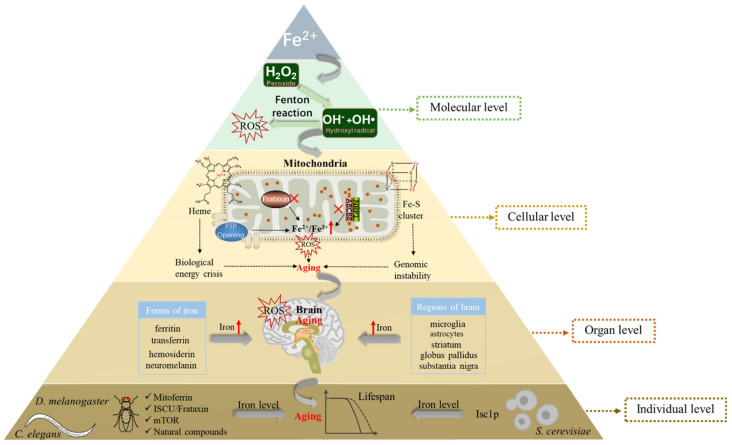
Relationship between iron and aging at the molecular, cellular, organ, and individual levels. At the molecular level, excessive iron in cells can participate in the Fenton reaction to generate ROS, resulting in damage to biological macromolecules and leading to aging. At the cellular level, defects in heme and iron–sulfur cluster biosynthesis in mitochondria with aging can lead to an energy crisis in organisms and instability of genomes. In addition, decreased Frataxin and Nrp1 levels or increased PTP levels promote the iron content of mitochondria, leading to aging through ROS. At the organ level, the age-associated increase in iron deposition varies between different brain regions and cell types, and then various molecular forms (ferritin, transferrin, hemosiderin, neuromelanin) of iron can deposit in the brain, resulting in brain aging. At the individual level, model organisms (*S. cerevisiae*, *C. elegans*, *D. melanogaster*) can regulate the iron level of the body through different mechanisms (such as mitoferrin, ISCU/Frataxin, mTOR, natural compounds, Isc1p), eventually leading to individual aging. The red fork represents the loss of function; The red upward arrow represents the increase in content. ROS, reactive oxygen species; Nrp1, neuropilin-1; ABCB8, mitochondrial transporter ATP-binding cassette B8; PTP, permeability transition pore; ISCU, iron–sulfur cluster (ISC) assembly machinery central protein; Isc1p, inositolphosphosphingolipid phospholipase C.

Reactive oxygen species (ROS) are chemically reactive substances containing oxygen, including superoxide (O^2−^), free radicals (HO^−^ and RO^−^), and peroxides (H_2_O_2_ and ROOH) [33]. Iron can participate in the formation of ROS in cells, which eventually leads to cytotoxicity. The Fe^2+^ and hydrogen peroxide in the organism can oxidize various substrates and cause biological damage, simultaneously producing hydroxyl radicals and a high oxidation state of iron. This reaction is called the Fenton reaction (Figure 2). The ability of iron to exchange single electrons with many substrates can lead to the generation of ROS, oxidative stress, lipid peroxidation, and DNA damage. These results will lead to genome instability and DNA repair defects, which ultimately damage cell vitality and promote programmed cell death [34]. In addition, iron and iron-containing complexes (such as heme or iron–sulfur clusters) are also necessary for ROS-producing enzymes [35]. It can be seen that the iron content in the body is particularly important for maintaining the level of ROS in cells.

### 3.2. Iron Accumulation in the Mitochondria and Aging

Mitochondria are active participants and are mechanistically linked to the unique biology of senescence [36]. The free radical theory of aging proposes that the cause of aging lies in the production of ROS at the level of the mitochondria, which over time causes extensive mitochondrial and cellular dysfunction [10]. Recent studies in yeast and mammals have shown that iron in mitochondria increases with age, especially under cell stress, which may be the potential cause of age-related mitochondrial dysfunction [37,38].

Iron in mitochondria is mainly used for the synthesis of heme or iron–sulfur clusters [39]. Frataxin is a response gene of Friedreich’s ataxia, that is necessary for the synthesis of heme or iron–sulfur clusters and plays an important role in the iron homeostasis of mitochondria [40]. When Frataxin is insufficient, iron will accumulate in the mitochondria, causing oxidative stress and mitochondrial dysfunction, eventually leading to cell apoptosis or aging [41,42]. In *Drosophila*, Frataxin-deficient flies are highly sensitive to increased dietary iron uptake, while their mitochondrial function can be restored by inhibiting iron uptake via mitoferrin [43]. New research has found lactoferrin ameliorates pathological cardiac hypertrophy, potentially by improving the mitochondrial quality related to mitochondrial dynamics, thus reducing mitochondria-dependent apoptosis [44].

Altered mechanisms of mitochondrial iron transport cause an accumulation of mitochondrial iron, which causes the decay of mitochondrial structural components, such as proteins, lipids, and nucleic acids. Recent studies have shown that the mitochondrial transmembrane protein Neuropilin-1 (Nrp1) and its interaction factor the mitochondrial transporter ATP-binding cassette B8 (ABCB8) play a critical role in iron accumulation and senescence in endothelial cells. The absence of Nrp1 reduces the protein level of ABCB8, which leads to the accumulation of iron in the mitochondria, inducing iron-dependent oxidative stress and aging [45]. Another study suggests that there is an increase in the susceptibility of the permeability transition pore (PTP) which may cause cellular degeneration via apoptosis or necrosis [46]. In yeast, cysteine-mediated iron deficiency is the main cause of aging-related mitochondrial dysfunction [37]. The loss of vacuolar/lysosomal acidity is an early event during aging that has been linked to mitochondrial dysfunction. Increased iron uptake may salvage the phenotype of mitochondrial dysfunction in yeast *vma* mutants, which have lost vacuolar acidity due to genetic disruption of the V-ATPase proton pump [47].

Mitochondrial dysfunction during aging is manifested by multiple defects in heme and iron–sulfur cluster biosynthesis in mitochondria with the increase of age, which may lead to an energy crisis of organisms and instability of genomes [48,49]. On the one hand, during the aging process of human fibroblasts, heme deficiency can selectively reduce the expression and activity of cytochrome C oxidase (complex IV), which is the terminal oxidase in the mitochondrial electron transport chain [50]. Therefore, it can be confirmed that age-related heme deficiency leads to impaired mitochondrial energy production by inhibiting complex IV. On the other hand, it was identified that there was a link between the defect of iron–sulfur cluster biosynthesis and age-related genomic instability in yeast [48]. Yeast cells will lose mitochondrial DNA (mt DNA) along with aging, which destroys the stability of the genome and leads to the reduction of mitochondrial membrane potential, finally leading to mitochondrial dysfunction. In aged muscle, cellular and mitochondrial iron homeostasis is perturbed, as reflected by altered levels of mitoferrin and frataxin, which might contribute to loss of mtDNA stability [51]. A recent study has demonstrated that lipoic acid supplementation may be considered as an adjuvant against mitochondrial damage and cognitive decline related to aging and neurodegenerative disorders [52]. Collectively, the above results indicated that iron was closely related to mitochondrial aging, therefore iron regulation may represent a possible target for aging interventions.

### 3.3. Iron Accumulation and Brain Aging

Ferrosenescence mainly occurs in the nervous system, which is closely related to the excessive deposition of iron ions in the brain [32]. The brain plays a unique role in iron metabolism because of the following characteristics: First, the brain resides behind the vascular barrier, which limits its access to plasma iron. However, little is known about the mechanism of iron release into the brain or the regulation of the transport mechanism. Insights into this transport mechanism could be crucial for understanding how an excess of iron can accumulate in the brain in many neurodegenerative diseases. Second, the iron concentration varies greatly between different brain regions and cell types. Autopsy analysis showed that the total iron concentration in the substantia nigra and globus pallidus of the basal ganglia increased with age [53]. Ashraf and colleagues found that the ratio of iron to microglia in an aging brain is elevated, especially in the basal ganglia, whereas the ratio of iron to astroglia is low in the striatum but elevated in the substantia nigra and globus pallidus. During aging, the astrocytes are vulnerable and susceptible to iron accumulation and oxidative damage [54]. In addition, various molecular forms of iron (ferritin, neuromelanin, transferrin, hemosiderin) between neurons and glial cells can be observed during aging [13,55]. Young individuals had relatively high levels of heavy-chain (H) ferritin compared to light-chain (L) ferritin; However, H and L-ferritin in the frontal cortex, caudate nucleus, putamen, substantia nigra, and globus pallidus are increased with age [56]. Notably, neuromelanin is the most prominent iron storage site in the substantia nigra and locus coeruleus, and its level will increase with age [57]. In short, the accumulation of iron will increase with individual aging in the nervous system.

As the mechanism of brain aging is very complex, it has not been fully understood. Nevertheless, increasing evidence shows that the damage to macromolecules and cell components caused by the destruction of cell redox balance and the imbalance of metal homeostasis is part of the cause of brain aging [58,59]. During brain aging, iron is partially transformed from its stable and soluble form (ferritin) to hemosiderin and other iron-containing hydroxides with higher reactivity, leading to neurons being susceptible to oxidative stress [60]. The transcription factor NF-E2-related factor 2 (Nrf2) is a central regulator of cellular antioxidant and detoxification responses. Studies have shown that the knockout of Nrf2 can reduce the level of iron transporter 1 (FPN1) in brain microvascular endothelial cells, thus reducing brain iron deposition and alleviating age-related motor dysfunction in aging mice [61]. Furthermore, recent evidence has suggested that iron accumulation, as observed in neurodegenerative disorders, hinders autophagy, which might play a part in iron-induced neurotoxicity. Rapamycin, by inducing autophagy, was able to ameliorate iron-induced cognitive impairments. These findings support the use of rapamycin as a potential neuroprotective treatment against the cognitive decline associated with neurodegenerative disorders [62]. Thus, a better understanding of the effect of iron homeostasis on brain aging may provide important insights into a better understanding of age-associated diseases.

### 3.4. Iron Metabolism and Life Expectancy

Iron content increases significantly with age, resulting in increased ROS production. This harmful process is not only limited to the central nervous system but also reflected in the whole individual, specifically in the length of their lifespan. In many model organisms such as worm, fly, and yeast, increasing antioxidant level [63] or inhibiting iron level by using iron chelators or using genetic manipulation to regulate iron metabolic processes [64,65] has been proved to prolong the lifespan.

The first evidence that inhibiting iron absorption prolongs lifespan was demonstrated in *Drosophila* [64]. By feeding male flies with a high-iron diet and tea extract, the results show that increasing dietary iron can shorten the lifespan, while dietary tea can prevent age-related iron accumulation. It has been reported that galloyl in tea polyphenols can inhibit iron-binding [66]. In addition, polyphenol compounds in tea extract not only inhibit iron absorption but also have a direct antioxidant effect, both of which may lead to prolonging the lifespan of *Drosophila*. A new study has shown that green tea may increase the lifespan of fruit flies by regulating mitoferrin and reducing mitochondrial iron [66]. As mitoferrin is required for iron to enter the mitochondria, reducing the mitoferrin level through RNAi treatment in the N2 wild-type strain can extend the lifespan by 50% to 80% in *C. elegans* [67]. Another finding indicates that iron-starvation-induced mitophagy as a protective mechanism against mitochondrial stress mediates lifespan extension in *C. elegans* [68]. Similarly, increased dietary iron intake significantly accelerates age-related protein aggregation in the worm and negatively affects organismal longevity [69].

In addition, many natural products and drugs prolong lifespans by seemingly completely different mechanisms, but most of them can chelate iron, so we can reasonably speculate that the life-prolonging mechanism of these compounds can be attributed to the reduction of the iron level in the cell. For example, curcumin is a strong iron-chelating agent. Animals fed curcumin have a decline in liver ferritin [70]. Mice fed 0.2% of curcumin in the diet become iron deficient [71]. It is interesting that curcumin and its metabolite tetrahydrocurcumin increase the average lifespan in at least three model organisms: *C. elegans*, *Drosophila*, and mice [72]. Ibuprofen can prolong the lives of many organisms [73]. Similarly, ibuprofen chelates iron, in this way, prevents oxidant lung injury [65]. Moreover, zebrafish have also shown that increased cellular iron concentration has negative health impacts that can be potentially ameliorated by iron chelation [74].

In addition to extending lifespan by chelating iron, genetically manipulating iron-related genes can be applied to alter iron levels. It is known that the inositolphosphosphingolipid phospholipase C (Isc1p) of *S. cerevisiae* belongs to the neutral sphingomyelinase family. The microarray analysis shows that Isc1p lacks an up-regulated iron regulator, resulting in elevated iron levels. Cells lacking Isc1p also displayed a shortened chronological lifespan which may attribute to the change in iron level [75]. Yeast MET18 is a subunit of the cytosolic iron-sulfur (Fe/S) protein assembly (CIA) machinery which is responsible for the maturation of Fe/S proteins. MET18 deficiency shortens the replicative lifespan of yeast by inhibiting catalase activity [76]. Studies have shown that calorie restriction can improve life expectancy because it can reduce ROS production and cell iron [77]. Apart from this, it was found that ISCU-1, the *C. elegans* ortholog of the evolutionarily conserved iron–sulfur cluster (ISC) assembly machinery central protein ISCU, suppressed longevity and stress response [78]. Specifically, ISCU-1 can accelerate aging of the intestine. Moreover, the Nrf2 transcription factor SKN-1 and a nuclear hormone receptor NHR-49 as the downstream factors of ISCU-1 have been identified. In addition, a mitochondrial outer membrane protein phosphatase PGAM-5 appears to link ISCU-1 to SKN-1 and NHR-49 in lifespan regulation. Frataxin can participate in the formation of the core complex NSF1/ISD11/ISCU, and its overexpression may contribute to the assembly of iron–sulfur clusters [79], thus inhibiting iron retention. In mice model of frataxin-depleted neurons, administration of fusion protein (TAT-MTScs-FXN) was able to produce a significant lifespan increase [80]. Genetic studies showed that the lack of Frataxin in *C. elegans* would shorten the lifespan [81], while the overexpression of Frataxin in *Drosophila* prolonged the lifespan [82], thus establishing the relationship between Frataxin and lifespan.

Moreover, in nematodes, by inhibiting lipid peroxidation or limiting iron retention, age-related cell death can be alleviated, thus the lifespan and healthspan can be significantly prolonged [83]. It is well known that the lifespan of a variety of model animals can be extended by inhibiting the mammalian target of rapamycin (mTOR), the important regulator of cell growth and proliferation. Studies have shown that mTOR plays a vital role in the body’s iron reserve. Specifically, activation of mTOR leads to an increase in body iron levels, which in turn activates mTOR. Therefore, one of the mechanisms inhibiting mTOR in prolonging lifespan may be through inhibiting body iron levels [84]. In addition, ferroptosis is an iron-dependent, nonapoptotic programmed cell death. A recent study indicated that the senolytic drug JQ1 can eliminate senescent cells via ferroptosis, suggesting ferroptosis as a new mechanism of senolytic therapy [85]. The above research implies that inhibiting iron levels may be one of the new ways to delay aging.

In short, at the molecular level, excessive iron in cells can generate a large number of reactive oxygen species (ROS) in the Fenton reaction, finally leading to aging. At the cellular level, mitochondrial iron levels change with aging, which in turn leads to mitochondrial dysfunction, causing further aging. Furthermore, at the organ level, iron concentration can increase in different areas and cell types of the brain, and various molecular forms of iron (ferritin, neuromelanin, transferrin, hemosiderin) can deposit in the brain, which together results in brain aging. Ultimately, at the individual level, model organisms such as *S. cerevisiae*, *C. elegans*, and *D. melanogaster* regulate the iron content of the body through different mechanisms, eventually leading to individual aging (Figure 2).

## 4. Iron Metabolism and Neurodegenerative Diseases

Currently, many studies have shown that metals are related to the pathogenesis and mechanisms of nervous system diseases [86,87]. There is enough evidence to prove that iron is indeed a key factor in neurodegenerative diseases [12]. In recent years, there has been increasing evidence that many neurodegenerative diseases are related to the excessive iron content in the brain. Progressive iron accumulation in the brain may produce free radicals via the Fenton reaction, promoting the occurrence of neurodegenerative diseases such as Alzheimer’s disease, Parkinson’s disease, and other neurodegenerative disorders.

### 4.1. Iron Metabolism and Alzheimer’s Disease

Alzheimer’s disease (AD) is a slow, progressive neurodegenerative disease involving the loss of cortical and hippocampal neurons, leading to impairment of cognitive functioning, including memory, language, and executive functioning [88]. AD has two pathological features: aggregation of Aβ, a major component of extraneuronal senile plaques (SPS), and hyperphosphorylation of tau, a microtubule-associated protein that forms intracellular neurofibrillary tangles (NFTs) [88]. Metal iron homeostasis disorders with redox activity may be related to the neuropathology of Alzheimer’s disease. With the development of iron quantification technology, especially the magnetic resonance imaging (MRI) method, it has been shown that the iron level in the brain of AD patients increases with age [89]. In addition, the application of other advanced technologies, such as inductively coupled plasma mass spectrometry (ICP-MS) analysis results shows that plasma iron is significantly reduced in patients with Alzheimer’s disease [90], which is speculated to be caused by excessive accumulation of iron in the brain. A recent analysis shows that iron levels and related redox activities in the cortex and cerebellum were increased in patients in the preclinical stage of AD [89]. As the Cisd2 gene encodes the CDGSH iron–sulfur-domain-containing protein 2, Cisd2 overexpression attenuates AD pathogenesis by guaranteeing mitochondrial quality and synaptic functions [91]. Therefore, brain iron accumulation has become a major pathological feature of the early onset of AD. To a certain extent, iron can act as a promising new target in AD for neuroprotection.

**Figure 3:**
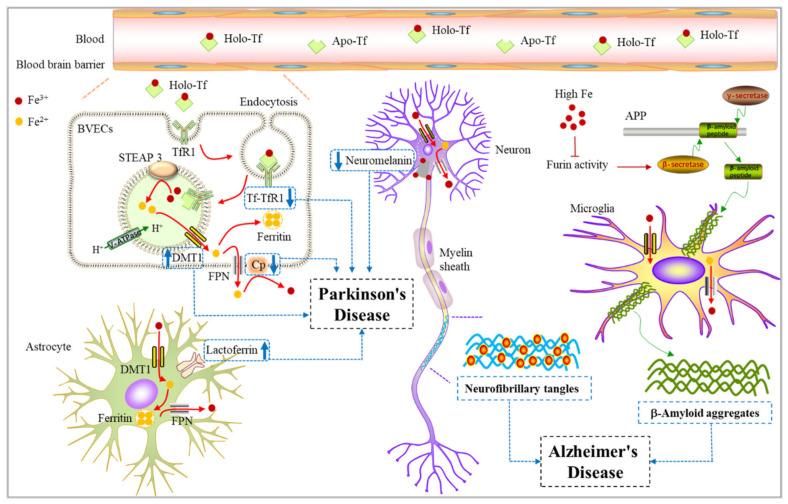
Relationship between iron and Alzheimer’s disease/Parkinson’s disease. Fe^3+^ can be absorbed and utilized by astrocytes, neurons, oligodendrocytes, and microglia in the brain. When the iron-related proteins change in neurons, the iron homeostasis is disturbed, leading to neurodegenerative diseases. Upregulation of some iron-storage proteins such as lactoferrin and transferrin increased expression of DMT1 in dopamine neurons. Both ceruloplasmin dysfunction and a decreased level of neuromelanin can cause an accelerated onset of Parkinson’s disease. Alzheimer’s disease is characterized by the formation of Aβ plaques and NFTs in the brain. The increase of intracellular iron level promotes β-secretase and γ-secretase activities to enhance the amyloid production pathway, and finally causes Aβ aggregation around microglia. In addition, iron also binds to tau, affecting its phosphorylation and inducing hyperphosphorylated tau tangles in neurons. Aβ aggregation and neurofibrillary tangles together lead to the occurrence of AD. Apo-Tf, Apo-transferrin; Holo-Tf, Holo-transferrin; BVECs, brain vascular endothelial cells; DMT1, Divalent metal-ion transporter 1; NFTs, Neurofibrillary tangles.

In terms of the regulation of iron in the pathogenesis of Alzheimer’s disease, most scholars believe that the homeostasis of zinc, copper, and iron is related to the misfolding process of amyloid (Aβ), amyloid precursor protein (APP), and hyperphosphorylated tau, which finally leads to neuronal oxidative stress [92] (Figure 3).

There is a close connection between iron homeostasis and the pathology of AD. It has been reported that the translation of amyloid precursor protein APP is directly regulated by cellular iron levels [93]. Most of the APP is cleaved by a nonamyloid production pathway. APP is first cleaved by α-secretase to produce sAPPα, which is then cleaved by γ-secretase to release the N- terminal fragment p3, leaving the intracellular domain of APP on the membrane. Under pathological conditions, APP passes through the neurodegenerative pathway of amyloid. APP can be cut by β-secretase and then cut by γ-secretase to produce Aβ [94]. The formation of Aβ and its accumulation in the brain can be weakened by stimulating α-secretase. Proteolytic activation of inactive forms of α -secretase and β -secretase is regulated by furin. The transcription of furin is regulated by intracellular iron concentration. Excessive iron leads to the reduction of furin concentration, thus increasing the activity of β-secretase and enhancing the amyloid production pathway. In contrast, iron deficiency increases the activity of furin, which enhances α-secretase and stimulates the nonamyloid production pathway [95]. Iron may regulate APP processing through IRPS, interacting with the hypothetical IRE in the 5′- untranslated region of APP mRNA [93]. The translation of APP may therefore be up-regulated under the condition of iron excess, increasing the amount of APP available to enter the amyloid production pathway, thereby resulting in the aggregation of Aβ peptide [93]. In addition to causing the accumulation of Aβ peptide, iron also binds to tau, affecting its phosphorylation and inducing hyperphosphorylated tau aggregation [96]. Accumulating hyperphosphorylated forms of tau have been demonstrated to be possible primary drivers of AD and play a key role in promoting neurotoxicity and neuronal loss [96]. In summary, iron can affect the progression of Alzheimer’s disease by regulating amyloid (Aβ), amyloid precursor protein (APP), and hyperphosphorylated tau (Figure 3).

The congenital iron overload disease, hereditary hemochromatosis (HH), is caused by mutation of the HFE gene [97]. The association between HH and AD has become increasingly apparent in recent years. HFE is expressed by reactive astrocytes as well as neurons in the brains of AD patients. The induction of HFE in the AD brain by stress factors, such as cells serum deprivation, menadione and beta-amyloid, were determined using BV-2 cells. The labile iron pool was consistently decreased when HFE expression increased. These data provide insight into the induction of HFE in AD and indicate that HFE expression may be a protective function to limit cellular iron exposure during cell stress [98]. Gene variants involved in iron homeostasis such as the HFE gene (His63Asp and Cys282Tyr variants) have been associated with a higher risk for AD. In single nucleotide polymorphisms (SNPs), the HFE 282Y allele yielded a three-fold risk reduction in the whole cohort of patients [99]. On the other hand, some studies have shown that C282Y and H63D HFE variants increase the risk for and severity of AD, especially in synergy with polymorphisms in the Tf gene [100]. An increased frequency of the transferrin C2 subtype was also noted in AD patients compared with age-matched controls [101]. The C2 variant, combined with the presence of the HFE mutation, increases the risk of AD fivefold [102]. To date, the relationship between HFE mutations and Alzheimer’s disease needs further investigation.

### 4.2. Iron Metabolism and Parkinson’s Disease

Parkinson’s disease (PD) is the second most prevalent neurodegenerative disorder worldwide, and its pathological feature is the degeneration of dopaminergic neurons in the dense part of the substantia nigra [103]. Iron can participate in the formation of oxygen free radicals and the induction of lipid peroxidation in the system due to its unique chemical properties, which are closely related to the PD process [104]. The results of the histochemical analysis showed that the iron deposition level in the substantia nigra of patients with PD was increased compared with that in the control group [105]. Quantitative analysis of brain iron content in PD patients using Mossbauer spectroscopy, atomic absorption, and atomic emission spectroscopy as well as colorimetric analysis showed that excessive iron shifted the Fe^3+^/Fe^2+^ ratio from 2:1 in the substantia nigra of normal people to 1:2 in the substantia nigra of PD patients. This shift to the more toxic form of iron may lead to the generation of hydrogen peroxide-derived reactive hydroxyl radicals through the Fenton reaction and promote the pathological process of Parkinson’s disease. At the same time, elevated iron was also observed in PD animal models induced by 6- hydroxydopamine (6-OHDA) and MPTP. However, the use of iron chelator or genetic manipulation of iron-related genes could prevent neuronal death in these models [106,107]. Furthermore, feeding the neonates of mice with a high-iron diet for 24 months resulted in a significant decrease in the number of TH+ dopaminergic neurons [108]. These studies have indicated that elevated iron may play an important role in the pathogenesis of Parkinson’s disease.

The cause of the accumulation of total iron in the substantia nigra of patients with PD is not well known, but several possible factors have been proposed, such as blood–brain barrier dysfunction [109,110]; upregulation of some iron-storage proteins such as lactoferrin [111] and transferrin [112,113]; Increased expression of DMT1 in dopamine neurons [114]; ceruloplasmin dysfunction and so on (Figure 3).

The blood–brain barrier (BBB) generally protects the brain from plasma iron influx. One potential source of increased iron is from peripheral influx through a disturbed or open BBB in the substantia nigra. In a previous study, researchers used radiolabeled verapamil hydrochloride and positron emission tomography (PET) in PD patients and age-matched healthy controls. Verapamil is a specific substrate for the P-glycoprotein (Pgp) multidrug resistance system in the cell membrane. Pgp functions as an efflux pump, and verapamil does not cross the BBB. The results showed that the uptake of verapamil in the midbrain of PD patients was higher, while the control group had no uptake. The reason is that the BBB’s efflux pump system (Pgp) cannot work properly in certain brain areas of PD patients, so that serum iron can enter the brain [110]. Therefore, blood–brain barrier dysfunction may be one of the causes for the accumulation of iron in the brains of patients with PD.

Some iron-storage proteins, for instance, lactoferrin, are closely related to PD. Studies have shown that human lactoferrin produces a neuroprotective effect and probably stimulates neuroregeneration under conditions of MPTP toxicity in the animal model of PD [115]. In addition, the transferrin/transferrin receptor transport pathway is also connected to neuronal iron uptake. For example, after MPTP was injected into the monkey brain to induce PD, the expression of transferrin receptor (TfR) in dopaminergic neurons and the substantia nigra of PD rats damaged by 6-OHDA was decreased [112]. Moreover, transferrin has been shown to decrease in the substantia nigra of PD by 35% [116]. Therefore, the analysis of these proteins in PD patients may provide important clues to clarify the mechanism of increased iron content in the brain of the patients.

Elevated iron concentrations in the substantia nigra might result from the manipulation of genes relevant to iron transport and binding. One example is that the haplotype (C alleles of 1254T and IVS4 + 44C/A polymorphisms) occurred at greater frequencies in PD subjects compared with that of control, suggesting that CC haplotype in DMT1 gene is a possible risk factor for PD in this Han Chinese population [117]. In addition, mice with mutant DMT1 with gene loss of function were partially resistant to 6-OHDA and MPTP-induced toxicity, further supporting the pathogenic role of DMT1 in iron-related neurodegeneration [114,118].

Both results in animal models [114] and patients [119,120] with PD have shown that ceruloplasmin dysfunction may be related to the disease. The decrease in ceruloplasmin iron oxidase activity and increase in copper concentration in the cerebrospinal fluid of PD may lead to increased mobilization of reactive iron and oxidative stress in the brain of the patient. Several missense mutations in genes encoding ceruloplasmin have been reported in PD patients, which may affect the mobilization of active iron [121], further indicating that ceruloplasmin iron oxidase may affect the pathogenesis of PD. In the substantia nigra, iron is stored in neuromelanin. Under normal circumstances, 50% of neuromelanin (NM) is saturated with iron. Neuromelanin can act protectively by chelating redox-active iron in the cytosol of neurons [122]. Previous work in patients with PD has shown that the iron content in neuromelanin was significantly reduced, increasing the content of redox-active iron (unstable Fe^2+^) in the substantia nigra [123].

The protein aggregation is mainly composed of highly ubiquitinated α-synuclein in the form of Lewy bodies, which is a pathological sign of PD. Lewy bodies are often found in the substantia nigra and are also present in neurodegenerative diseases, such as dementia with Lewy bodies [124]. Previous studies have shown that iron can stimulate intracellular α -synuclein and ubiquitin aggregation in PD patients [125]. There are toxic feedback loops of iron, reactive oxygen species, and α -synuclein in PD patients. ROS and iron will increase each other’s levels, which will put the cells under oxidative stress and lead to the aggregation of α-synuclein. α-synuclein can in turn increase ROS and iron by inducing mitochondrial dysfunction and iron reductase activity. These effects are transmitted to neighboring neurons in a toxic feedback loop, which leads to PD pathology [126].

Heme oxygenase-1 (HO-1) is an inducible enzyme known for its anti-inflammatory, antioxidant, and neuroprotective effects. However, increased expression of HO-1 during aging and age-related neurodegenerative diseases has been associated with neurotoxic ferric iron deposits. It is highlighted that microglial HO-1 overexpression contributes to neurotoxic iron accumulation, creating deleterious effects in aged mice exposed to an inflammatory insult [127]. Plasma HO-1 levels may be a promising biomarker of early PD. A recent study found that plasma HO-1 levels were significantly elevated in PD patients, predominantly those with early-stage PD, compared with controls [128]. Besides, elevated HO-1 correlates with increased brain iron deposition measured by quantitative susceptibility mapping and decreased hemoglobin in patients with Parkinson’s disease [129].

Briefly, iron can play an important role in the regulation of the activity of neurons in a variety of ways. Understanding the mechanism of iron in the regulation of PD is expected to provide certain clues for the subsequent treatment of Parkinson’s disease.

## 5. Other Iron-Related Neurological Diseases

Some less common neurodegenerative diseases, mainly include a series of neurodegeneration with brain iron accumulation (NBIA), such as Friedreich’s ataxia (FRDA), Aceruloplasminemia, Neuroferritinopathy, and Pantothenate kinase-associated neurodegeneration (PKAN).

Friedreich’s ataxia (FRDA) is an autosomal recessive neurodegenerative disorder that principally affects the heart and the nervous system [130]. The pathogenic mechanism of Friedreich’s ataxia at the gene level is relatively clear. The GAA trinucleotide repeat sequence amplified in the first intron of the frataxin gene is the most common mutation in the frataxin gene, inducing a substantial reduction in the concentration of the mitochondrial protein frataxin [131]. Conceptually, FRDA is a disorder of iron distribution rather than simply an overload. Iron accumulates in the mitochondria in FRDA and animal models based on frataxin deficiency [15].

Aceruloplasminemia is caused by a mutation in the gene coding for ceruloplasmin protein [132]. Ceruloplasmin (Cp), also known as copper oxidase, is a blue-looking copper (Cu) glycoprotein. It has six compact domains that can bind to six Cu atoms. Cp carries 40–70% of Cu in plasma and plays important roles in Cu transport, iron (Fe) regulation, free radical scavenging, and antioxidant processes [133]. Among the numerous NBIA, Aceruloplasminemia has the highest level of iron accumulation and it has the final impact on the liver, pancreas, retina, and many brain regions, including basal ganglia and cortex [134].

Neuroferritinopathy is a rare autosomal dominant hereditary disease that is caused by a mutation in the L-ferritin gene. It can increase the level of free redox-active iron, and thus make neuron cells undergo oxidative stress [135]. The most common clinical manifestations are dyskinesia, behavioral abnormalities, and cognitive deficits., Iron deposition in the basal ganglia, abnormal ferritin and iron accumulation in the globus pallidus and substantia nigra, and low serum ferritin concentration were found in patients with Neuroferritinopathy [136].

Pantothenate kinase-associated neurodegeneration (PKAN) is caused by mutations in the gene encoding pantothenate kinase 2 (PANK2) which is a major genetic defect in NBIA [16]. PKAN is an autosomal recessive genetic disease characterized by dystonia and retinopathy pigmentosa in children and speech or neuropsychiatric defects in adults. In PKAN patients, the accumulation of iron in the globus pallidus is 3–4 times that of normal subjects [137].

## 6. Treatment Strategies Using Iron Chelators

Metal chelators can strongly bind metal ions to become stable and large molecular weight compounds, thus preventing metal ions from their action. It can therefore be used for detoxification. Iron chelation therapy may prevent iron-induced ROS, oxidative stress, and α-synuclein and Aβ aggregation, implying that iron chelation is a feasible neuroprotective approach for neurodegenerative disease and other nervous systems disease associated with abnormal iron metabolism [138].

Considering that iron deficiency can also cause harmful effects, unlimited removal of iron is undesirable. Potential therapeutic agents should promote the homeostasis of iron, rather than its complete removal. Iron chelators have some characteristics, such as being highly soluble and easily crossing the blood-brain barrier. In addition, they must have a high degree of specificity and selectivity for the chelation of iron ions [139]. It must also be noted that potential chelators can not only function by removing metal ions but also protect tissues by affecting other cellular mechanisms. For example, desferrioxamine has an additional neuroprotective effect by inducing hypoxia-induced transcription factor 1 DNA binding and erythropoietin transcription [140]. The characteristics and targeted diseases of several common chelating agents are described below in Table 1.

As mentioned above, with increasing evidence that iron homeostasis disorder could be one of the mechanisms of pathological deterioration in PD, the recovery of iron homeostasis in the brain may become a feasible target for a new therapeutic design. For example, in rat PD models, desferrioxamine can be used to protect dopaminergic neurons from death efficiently [141]. Considering that desferrioxamine is unable to travel across the BBB due to its size and hydrophilic nature, new research declared that compound Clioquinol improved motor and non-motor deficits in an MPTP-induced monkey model of PD through the AKT/mTOR pathway [144]. Many other-chelating agents have also been found, including deironiprone, which can protect dopaminergic neurons from neurodegeneration [151].

In addition to being used for the treatment of PD, deferoxamine is also the first metal chelator studied in AD patients. Studies have found that compared, with placebo and no treatment, treatment with this compound significantly reduces the clinical progression of dementia [142]. This manner avoids using a traditional iron-chelating agent, which is hydrophilic and difficult to pass through the BBB. In addition, deferoxamine also has a high affinity for other metal ions (aluminum, copper, and zinc). In other words, it has low selectivity for metal ions [152]. An advanced compound, PBT2, mainly binds to excess copper and zinc and possibly iron in the brain, thereby reducing the number of amyloid plaques and relocating these metal ions to the depleted cell and neuron compartments [145].

At present, there have been many experiments on the administration of deferiprone to Friedreich ataxia patients. One of the studies found that, compared with the control group, the iron content in the dentate nucleus of the deferiprone treatment group had been reduced [153]. In addition, the nervous system-related functions have also been significantly improved, such as manipulative dexterity, speech fluency, and ataxia gait, especially for young patients. Another study revealed when deferiprone and antioxidant idebenone were given to FRDA patients aged 8–25 years old, the combination had a stable effect on the normal function of the nervous system [154]. In short, suitable doses might be beneficial in younger patients with less severe disease, although more cases are needed for validation.

Many natural polyphenols, due to their ability to chelate metal ions, play an important role in the treatment of neurological diseases. Simultaneously, these polyphenols themselves have antioxidation and anti-inflammatory effects that can effectively alleviate the oxidative stress caused by metal ions [146]. For instance, curcumin chelates Cu and Fe transition ions, which play roles in AD pathology [147]. Epigallocatechin-3-gallate (EGCG) is the most abundant catechin found in the green tea plant. EGCG has the potential to combine with Aβ to protect nerves from damage because of its distinct antiamyloidogenic reactivity toward metal-Aβ species with a structure-based mechanism [146]. Besides, quercetin is a promising therapeutic candidate due to its bifunctionality as an antioxidant and chelating molecular [148]. Therefore, it is believed that natural polyphenols have great potential in the treatment of neurodegenerative diseases, implying that the development of more efficient natural polyphenols is critical in the future.

More recently, many novel chelating agents for the treatment of neurodegenerative diseases are emerging. A substance called pro-chelators must be induced by H_2_O_2_ to chelate metal ions, which can effectively reduce the oxidative stress caused by metals. Compared with traditional chelating agents, they can more effectively chelate metals and maintain intracellular metal homeostasis [155]. Some non-toxic lipophilic brain-permeable iron chelators provide potential therapeutic benefits for progressive neurodegenerative diseases, such as VK-28, HLA-20, and M30 [149]. Another promising compound, 6-methoxysalicylaldehyde nicotinoyl hydrazone (SNH6), showed low cytotoxicity, potent iron-chelation efficacy, significant inhibition of copper-mediated Aβ aggregation, alleviation of oxidative stress, as well as the effective donation of NAD^+^ to NAD-dependent metabolic processes (PARP and sirtuin activity). It significantly increased the median lifespan of *C. elegans*. Based on these benefits to the organisms, SNH6 has the potential to act as a multifunctional therapeutic agent for AD treatment [150].

In summary, iron is believed to be a novel target for pharmacological interventions in these disorders (Table 1). Reducing iron toward normal levels or hampering the increases in the iron of the brain is a promising therapeutic strategy for all iron-related neurodegenerative disorders.

## 7. Conclusions

Iron is a necessary trace metal element for organisms and is involved in many important physiological processes. However, iron is also a double-edged sword, which will produce toxic effects when overloaded. Therefore, the maintenance of iron homeostasis is particularly vital for the normal physiological function of the organism. Excess iron in the body can produce large amounts of ROS through the Fenton reaction, which in turn damages many macromolecular substances, such as lipids, proteins, and DNA. The increased mitochondrial iron level results in the accumulation of ROS, leading to mitochondrial dysfunction and eventually cell senescence. The accumulation of iron in the brain affects the normal function of the brain and leads to the occurrence of many age-related neurodegenerative diseases. However, many iron chelators can be applied to chelate excess iron to reduce iron levels for the treatment of diseases.

Nevertheless, we should carefully investigate whether iron overload is the cause or the result of aging. If iron overload is the cause of aging, iron chelators may be effective in the treatment and may delay aging. On the other hand, if iron overload is a consequence of aging, clarifying why iron accumulates with age will indicate the treatment of neurodegenerative diseases related to iron overload. In either case, the role of iron in cell senescence remains largely uncertain. Therefore, research on the mechanisms underlying this effect of iron metabolism on aging and aging-related neurodegenerative diseases still needs to be explored further.

## Figures and Tables

**Table 1:** Properties of common chelating agents, characteristics and targeted diseases.

Iron Chelating Agent	Characteristic	Targeted Diseases
Desferrioxamine	Induced HIF-1 DNA binding and transcription of erythropoietin in vivo; brain-impermeable; hydrophilic and high molecular weight; low selectivity for metal ions.	PD/AD [140,141,142]
Clioquinol	Brain-permeable; lipophilic; high toxicity.	PD/AD [143,144]
PBT2	high selectivity.	AD [145]
Natural polyphenols (curcumin/EGCG/quercetin)	Nontoxic; brain-permeable; anti-oxidation/inflammatory.	PD/AD [146,147,148]
Novel chelating agents (VK-28/HLA-20/M30/SNH6)	Low-toxicity; lipophilic; brain-permeable.	PD/AD [149,150]

## Data Availability

Not applicable.
